# Structural Basis of Rap Phosphatase Inhibition by Phr Peptides

**DOI:** 10.1371/journal.pbio.1001511

**Published:** 2013-03-19

**Authors:** Francisca Gallego del Sol, Alberto Marina

**Affiliations:** 1 Instituto de Biomedicina de Valencia, Consejo Superior de Investigaciones Científicas (IBV-CSIC), Valencia, Spain; 2 CIBERER, Valencia, Spain; HHMI, Massachusetts Institute of Technology, United States of America

## Abstract

Two-component systems, composed of a sensor histidine kinase and an effector response regulator (RR), are the main signal transduction devices in bacteria. In *Bacillus*, the Rap protein family modulates complex signaling processes mediated by two-component systems, such as competence, sporulation, or biofilm formation, by inhibiting the RR components involved in these pathways. Despite the high degree of sequence homology, Rap proteins exert their activity by two completely different mechanisms of action: inducing RR dephosphorylation or blocking RR binding to its target promoter. However the regulatory mechanism involving Rap proteins is even more complex since Rap activity is antagonized by specific signaling peptides (Phr) through a mechanism that remains unknown at the molecular level. Using X-ray analyses, we determined the structure of RapF, the anti-activator of competence RR ComA, alone and in complex with its regulatory peptide PhrF. The structural and functional data presented herein reveal that peptide PhrF blocks the RapF-ComA interaction through an allosteric mechanism. PhrF accommodates in the C-terminal tetratricopeptide repeat domain of RapF by inducing its constriction, a conformational change propagated by a pronounced rotation to the N-terminal ComA-binding domain. This movement partially disrupts the ComA binding site by triggering the ComA disassociation, whose interaction with RapF is also sterically impaired in the PhrF-induced conformation of RapF. Sequence analyses of the Rap proteins, guided by the RapF-PhrF structure, unveil the molecular basis of Phr recognition and discrimination, allowing us to relax the Phr specificity of RapF by a single residue change.

## Abstract 2

A structural and functional study shows the molecular mechanism of Rap protein inhibition by Phr signaling peptides, providing new insights into peptide recognition and discrimination in quorum sensing.

## Author Summary

In microorganisms, two component signaling systems are widely used to sense and respond to environmental changes, including quorum-sensing of Phr oligopeptides. Although the minimal machinery required for these systems comprises a sensor histidine kinase and an effector response regulator (RR), ancillary proteins, termed “connectors,” capable of modulating the activity of this machinery, are emerging as additional players in this complex signaling process. Rap proteins are archetypal connectors, able to modulate the activity of RRs either by dephosphorylating them or by physically blocking them. Rap proteins are themselves in turn inhibited by specific Phr peptides, adding an extra level of complexity, but how a Rap protein is regulated by its cognate Phr peptide remains unknown. To answer this question, we solved the structure of RapF, a Rap family member that blocks RR ComA, alone and in the complex with its inhibitory peptide PhrF. Our structural and functional results reveal that PhrF blocks the RapF-ComA interaction by an allosteric mechanism since the PhrF-RapF interaction induces a conformational change that is propagated to the the ComA binding site, disrupting it and triggering the dissociation of ComA from RapF. Using sequence analysis guided by our structure, we pinpointed sets of residues responsible for peptide anchor and specificity, respectively, and were able to relax RapF-Phr specificity simply by changing a single residue. Knowledge of these key residues and the Rap inhibition mechanism opens up the possibility of re-engineering Rap proteins, and paves the way to reprogramming signaling pathways for biological and biotechnological applications.

## Introduction

Bacteria communicate with each another and coordinate essential processes such as biofilm formation, sporulation, competence, virulence, or swarming motility in different ways. Quorum sensing is one of these mechanisms regulated by cell population density, and is mediated by self-generated extracellular signal molecules to allow the coordination of community-wide behaviors. Both Gram-negative and Gram-positive bacteria exploit quorum-sensing signaling, generally through different messenger molecules. In the former, acyl-homoserine lactones are the predominant signals, whereas the quorum sensing in the latter relies on the secretion and recognition of oligopeptides. These signaling peptides elicit a response either directly by interacting with their intracellular receptor after an importing process or indirectly by modulating the activity of a membrane-bound two-component sensor histidine kinase in the responder cell [1]–[3].

The RNPP family (named after its members: Rap/NprR/PlcR/PrgX) of quorum-sensing proteins comprises Gram-positive regulators, which bind directly to their signaling peptide in the receiver cell [4],[5]. Structural and functional data indicate that the members of this family share a similar architecture, which is composed of an N-terminal effector domain that interacts with the target and a C-terminal regulatory domain that recognizes the oligopeptide [4],^6^–^12^. Three (NprR, PclR, and PrgX) of the four RNPP family members present an effector domain, which adopts the characteristic DNA-binding helix-turn-helix (HTH) fold and exerts its activity by directly interacting with DNA [4],[8],[9]. In contrast, the N-terminal domain of Rap proteins folds as a 3-helix bundle that mediates their action by interacting with their targets [6],[7], which, in most cases, are the two-component signaling protein response regulators (RR). RNPP regulatory domains contain from five (PlcR) to nine (NprR) degenerated tetratricopeptide repeats (TPRs) [8]. TPRs are helical domains that mediate protein-protein interactions and the assembly of multiprotein complexes [13]. The TPR motif consists of 34 amino acid residues with a poorly conserved consensus sequence. Structurally, TPR motifs fold as two antiparallel α-helices, denoted helix A and helix B, which adopt a helix-turn-helix arrangement. Usually, several TPR motifs pack in a parallel fashion to generate a right-handed superhelix with an internal concave surface, mainly contributed by the residues in helices A [13]. For some RNPP family members, it has been shown that the recognition and binding of signaling peptides is mediated by the TPR [4],[8],[9],[11]. However, the binding of signaling peptides to each RNPP representative seems to have dissimilar effects since PlcR and NrpR are activated, but Raps and PrgX are inhibited by their corresponding oligopeptides [8],[12],[14]–[16].

Rap proteins have been exhaustively studied in *Bacillus subtilis*, which expresses 11 chromosomal- and five plasmid-encoded members 17–21. Several Rap proteins block the signaling mediated by the two-component system by interacting with RRs. However, two completely different ways of accomplishing this function have been reported for members of this family. One subset of Rap proteins, including RapA, RapB, RapE, RapH, and RapJ, displays phosphatase activity to their target RRs [6],[7],[15],[19],[21],[22]. The second subgroup, comprising RapC, RapF, RapG, RapH, and RapK, blocks the action of the target RR by a direct interaction with their DNA binding domain, and works as an anti-activator [6],[21],[23]–[25]. Interestingly, RapH possesses both activities [21]. The complexity of the Rap signaling system increases by the participation of the aforementioned regulatory oligopeptides. For Rap proteins, regulatory peptides are called Phr and their mature active form is a penta- or hexa-peptide generated from a ∼40 amino-acid precursor by means of a post-transcriptional export-import process [16],[22],[26]–[28]. Phr peptides are commonly linked to their target Rap proteins in such way that *phr* genes are situated immediately downstream of the genes encoding the Rap proteins to form rap-phr signaling cassettes, which are concurrently transcribed [3]; thus, the Phr peptide is named after the Rap protein. Eight (RapA, RapC, RapE, RapF, RapG, RapH, RapI, and RapK) of the 11 genome-encoded Raps in *B. subtilis* form rap-phr signaling cassettes. RapB is regulated by the RapC (PhrC) peptide, while RapD and RapJ remain as Phr orphan Raps [3],[16].

Recently, the tridimensional structure has been reported of two Rap family members, RapF and RapH, in complex with their RR targets, ComA (DNA binding domain) and Spo0F, respectively [6],[7]. These structures have revealed that RapF and RapH are structurally similar, but that they bind their cognate RR at distinct non overlapping sites, mainly localized in the 3-helix bundle N-terminal domain of both proteins [6],[7]. Since it is anticipated that Phr peptides are recognized by the C-terminal TPR domain [29], it has been proposed that the inhibition of Rap proteins by the signaling peptides could be mediated by Phr-induced conformational changes [6]. In order to demonstrate how Rap proteins inhibition is accomplished by Phr peptides, we determined the tridimensional structure of *B. subtilis* RapF alone and in complex with its cognate inhibitory pentapeptide PhrF. The structures show that the TPR domain of RapF recognizes and binds the PhrF peptide, and that six of the seven TPR motifs in this domain participate in the process. The sequence analysis of the Rap proteins guided by the free and RapF-PhrF structures allow us to identify critical positions in the Rap-Phr interaction and to unveil two types of residues responsible for mediating either peptide anchoring or peptide selectivity. The comparison of RapF-PhrF, RapF-free, and RapF-ComA reveals major movements in RapF induced by PhrF and provides a mechanistic insight into the molecular basis of Rap protein inhibition by signaling peptides.

## Results

### Overall Structure of RapF and RapF-PhrF Complex

**Figure 1:**
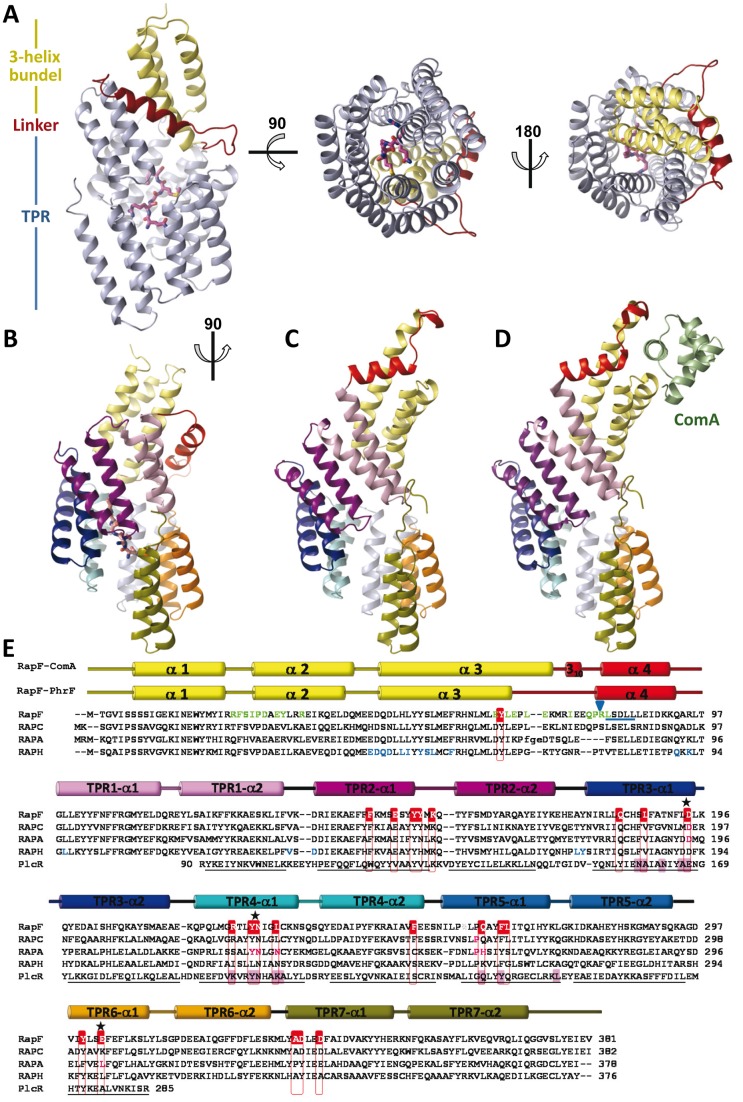
Structure of RapF-PhrF, RapF free, RapF-ComA, and structure-guided sequence alignment of RNPP family members. (A) Ribbon representations of RapF-PhrF complex in three orthogonal views. The 3-helix bundle is colored in yellow, the linker region in red, and the TPR domain in light blue. PhrF is shown in sticks rendering with carbon atoms colored in magenta. (B–D) Conformations of RapF. Ribbon models of the three forms of RapF, (B) PhrF complex, (C) free, and (D) ComA complex (Protein Data Bank entry 3ULQ), are shown in the same orientation with the 3-helix-bundle colored in yellow, the linker region in red, and the TPR1 in light purple, TPR2 in dark purple, TPR3 in dark blue, TPR4 in cyan, TPR5 in light blue, TPR6 in orange, and TPR7 in olive. In the RapF-ComA structure, ComA DNA binding domain is represented in ribbon and colored in pale green. (E) Sequences of four *B. subtili*s Rap family members and PlcR (TPR domain only) from *B. cereus* are aligned guided by the structures when were available (RapF, present study and PDB = 3ULQ; RapH, PDB = 3Q15; and PlcR, PDB = 2QFC). The secondary structure elements for RapF in complex with ComA and PhrF are represented above the sequence, named and colored as in (B) and (D). Black lines indicate the TPR helices as obtained from the PlcR structure. The residues interacting with PhrF are surrounded by red boxes and white lettering for RapF sequence. PlcR residues implicated in PapR binding are highlighted with pink shadows [4]. Residues highlighted in green and blue at RapF and RapH sequences, respectively, are implicated in the RR binding [6],[7]. Substitutions at the positions highlighted by purple lettering in the sequence of RapC and RapA abolished peptide binding [11],[19],[25]. Black starts indicate residues substituted in the present works that abolish or alter the peptide binding. The blue arrowhead points the trypsin cut position and the sequence obtained after Edman sequencing of the major tryptic fragment is underlined in blue.

In order to determine the molecular basis of RapF inhibition by Phr peptides, the X-ray structures of RapF alone and in complex with its inhibitory pentapeptide PhrF (QRGMI) were determined. The structures were solved using the anomalous signal of the selenium or platinum atoms for the apo or the PhrF complex structures, respectively (Table S1). The crystal asymmetric unit showed two molecules in the free RapF structure and one RapF molecule bound to one PhrF peptide in the structure of the complex. The structural models for the free and PhrF complex forms were refined to a final resolution of 2.25 and 3.1 Å, respectively (Figure 1A–1C; Table S1). Despite the limited resolution data for the RapF-PhrF complex, density maps of exceptional quality were obtained from the experiential phases and improved by density modification due to the high-solved content (75%) of the crystals (Figure S1). The RapF protein model, the PhrF peptide, and the contact described herein were clearly visible in these maps, except for the nine C-terminal residues (residues 376–381) where electronic density was absent, which reflects the elevated flexibility of this region.

As previously disclosed by the crystal RapF structure in complex with the DNA-binding domain of its RR target ComA [6], RapF was an all-helical protein consisting of two domains: a small N-terminal 3-helix bundle domain (residues 1–68) and a large C-terminal TPR domain (residues 98–370), both connected by a linker region (69–97) (Figure 1). The RapF-ComA structure showed that the 3-helix bundle, together with the linker region, formed the ComA binding surface (Figure 1D). A comparison of the free RapF and the RapF-ComA structure reveals that binding of ComA to RapF only promoted slight local conformational changes in RapF, which were mainly restricted to the RR recognition domain (the 3-helix bundle plus the linker region; 1–97) in order to bind the DNA binding helix of ComA (Figures 1C, 1D, and S2). The core TPR domains remained at the same position (root mean square = 0.66 Å for the superimposition of residues 98–380; Figure S2), which supports that RapF in solution presents an active conformation that is competent to bind ComA. Since RapF presented a similar conformation in the free and the ComA complex forms (Figure S2), here we discuss the conformational changes observed in the RapF-PhrF complex with regard to the structure of both RapF-ComA and RapF-free indistinctly. The RapF-PhrF structure reveals that the inhibitory peptide was bound to the TPR domain (Figure 1A). The most striking difference between the RapF structures was the relative disposition of the N- and C-terminal domains. When RapF was free or in complex with ComA, the 3-helix bundle domain was projected apart from the TPR domain, thus exposing the ComA binding surface. In contrast, this domain retracted and was laid on the TPR domain when PhrF was bound (Figure 1B–1D). The most affected structural elements by PhrF-induced movements were the C-terminal part of the α3 helix in the 3-helix bundle domain and the connected linker region. The 3-helix bundle α3 helix was five residues shorter in the RapF-PhrF structure. Moreover these residues, together with the 3_10_ helix of the linker region observed in the RapF-ComA structure, formed a long unstructured loop in the complex with the peptide (Figures 1B–1D).

### PhrF Binding Site

**Figure 2:**
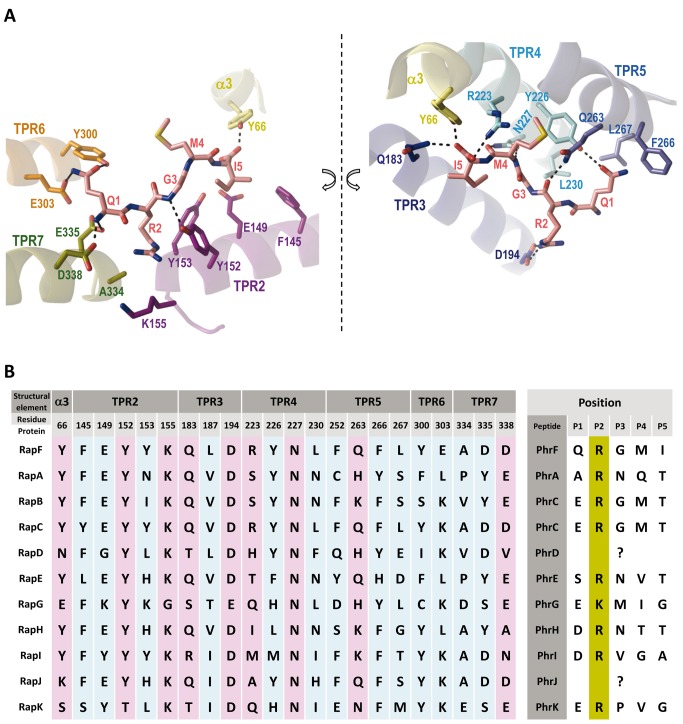
PhrF recognition by RapF. (A) Close view of the PhrF binding site that is presented in two halves dissected along the peptide axis for easier visualization. Colors are as in Figure 1B. Peptide interacting residues are shown in stick, labeled and colored with the carbon atoms as the corresponding structural element. PhrF is shown in sticks, labeled and colored with carbon atoms in pink. RapF-PhrF polar interactions are drawn as dashed black lines. (B) Peptide interacting residues in Rap proteins from *B. subtilis*. Residues for the 11 Rap proteins from *B. subtilis* corresponding to the RapF positions interacting with PhrF are aligned. Anchor and specificity residues are highlighted by magenta and light blue boxes, respectively. The numbers indicate amino acid positions of RapF. (Right) For each Rap protein the corresponding Phr Inhibitory peptide is shown. The conserved positive charged residue at position 2 is highlighted in mustard.

The RNPP family of Gram-positive quorum sensors presents a characteristic C-terminal TPR domain with seven TPR repeats for the structurally known Raps (RapF and RapH) (Figures 1E and S3) [30],[31]. The RapF TPR domain folded in a large superhelix to generate a pseudo circular structure that was closed in a wide channel (8,588 Å^3^, calculated by the CASTp software [32]) by the interaction of the terminal part of TPR7 (and the following C-terminal tail) with the initial part of TPR1 (Figure 1). As a channel, the generated ring-like structure was open on both faces (Figures S4, upper panel). PhrF placed on the concave side of the RapF TPR channel, similarly to that observed for PlcR and PrgX, two RNPP representatives, in complex with their regulatory peptides (PapR and cCF10, respectively) (Figures 1A, 1B, and S3) [4],[9]. Peptide binding to RapF induced a severe constriction in the TPR channel (volume lowered to 4,954 Å^3^) and a large displacement of the 3-helix bundle domain (see below), which partially closed one of the channel faces (Figures 1B, 1D, and Figures S4, lower panel). PhrF lies in an extended conformation, as described for several TPR-peptide complexes [13], and interacted with the residues of six of the seven TPR repeats (TPR2–TPR7) (Figures 1 and 2). Additionally, the interaction of Tyr66 from the 3-helix bundle with the PhrF carboxy-terminus was the unique contact between this domain and the peptide, and accounted for the requirement of the free terminal carboxylate group described for the PhrA inhibition of RapA [33]. A comparison of the peptide-free and bound RapF structures showed that TPR4–TPR7 generated a preformed site (minimum displacement between both structures) where the peptide was positioned (see below). The central part of the peptide main chain was fixed by polar interactions. In particular, the strictly conserved Asn in the RNPP superfamily (Figures 1E and 2), Asn227 in RapF, emerged from TPR4 to bind by a hydrogen bond with PhrF Met4, and a Rap family conserved TPR2 Tyr (Tyr152 in RapF) bound the main chain oxygen of PhrF Gly3 (Figures 1E and 2). The peptide adopted its extended conformation by pulling interactions from both the N- and C-terminal ends. The C-terminal PhrF oxygens interacted with Arg223 (TPR4) by a salt bridge, and with Gln183 (TPR3) and Tyr66 (3-helix bundle) by both hydrogen bonds. On the opposite end, Asp338 from TPR7 was salt-bridged with N-terminal PhrF nitrogen (Figure 2). The PhrF side chains, with the obvious exception of Gly3, established extensive contacts with the less conserved TPR residues in the Rap family. PhrF Gln1 was hydrogen-bound to Tyr226 (TPR4) and Tyr300 (TPR6), and came into hydrophobic contact with the side chains of Gln263 and Phe266 on TPR5 (Figure 2). The side chain of PhrF Arg2 was situated in a hydrophobic pocket formed by Tyr152, Lys155 (both from TPR2), and Ala334 (TPR7), which was closed by the conserved Asp194 (TPR3), which interacted with Arg2 via a salt bridge (Figure 2). Similarly, PhrF Met4 and Ile5 were inserted into the hydrophobic clefts generated by the side chains of Tyr226 (TPR3), Phe252 (TPR4), and Gln263 (TPR5) for Met4, and Phe145, Glu149, Tyr153 (all from TPR2), and Leu187 (TPR3) for Ile5, with Arg223 (TPR4) participating at both sites (Figure 2).

### Decoding Phr Binding and Specificity

In accordance with the contacts described in the previous sections, the residues involved in Phr peptide binding and recognition were grouped into two sets. The first set should include those interactions anchoring the peptide onto the TPR domain to ensure that the peptide sets the protein in a correct orientation and to guarantee the extended conformation. The second set should correspond to the variable residues in the Rap family, which confer specificity among peptides.

**Figure 3:**
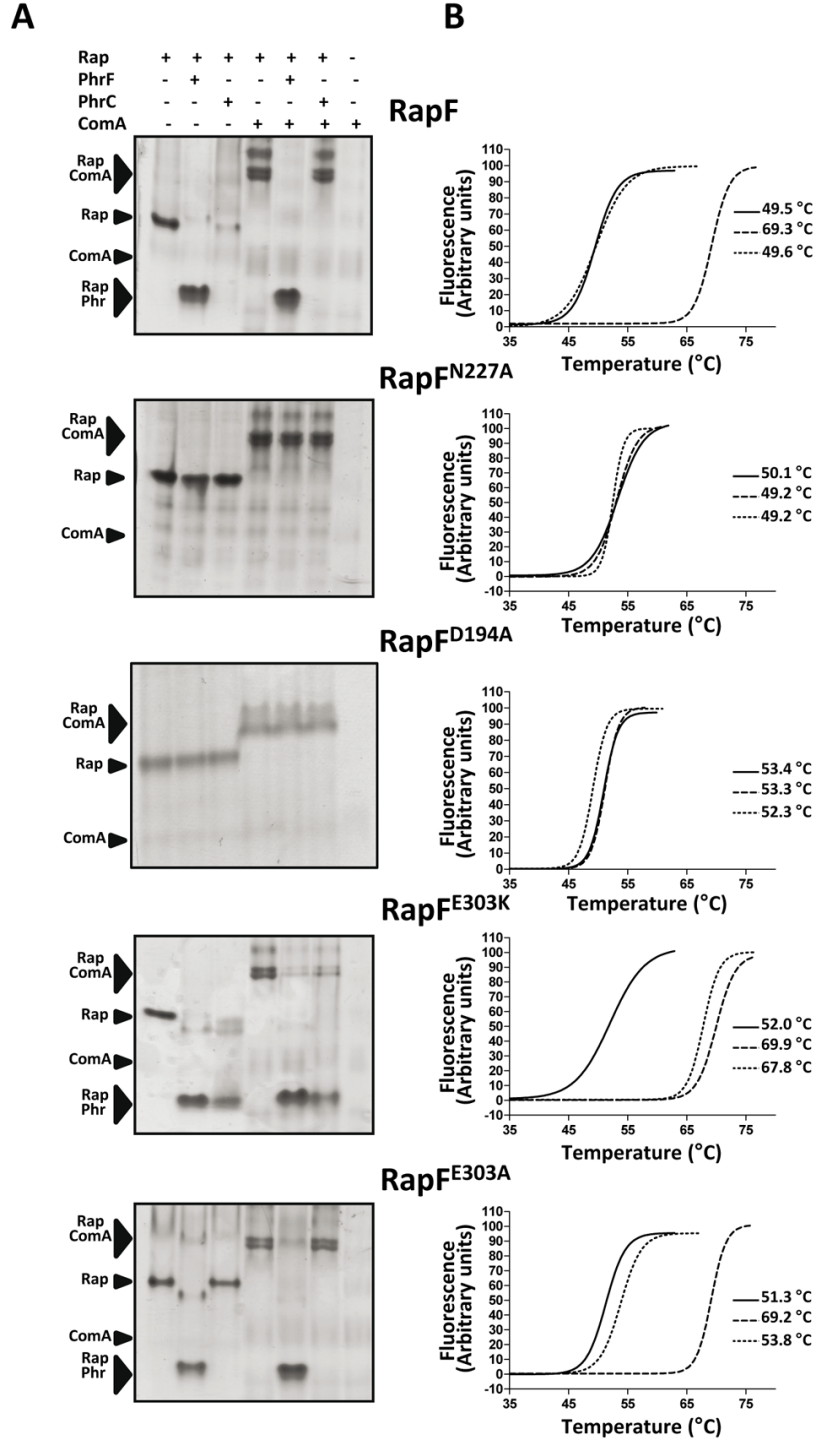
In vitro analysis of RapF and RapF mutants interaction with peptides and ComA. (A) Native gel assays. The interaction of native RapF and the RapF mutants Asn227Ala (RapF^N227A^), Asp194Ala (RapF^D194A^), Glu303Lys (RapF^K303A^), and Glu303Ala (RapF^E303A^) with the inhibitory peptides PhrF and PhrC and the RR ComA were analyzed by Native-PAGE and Coomassie-stained. The positions of the individual proteins and the peptide-Rap or ComA-Rap complexes are indicated by black arrowheads and labeled. (B) Thermal-shift assays. Representative thermal denaturation curve profiles of wild type and mutant variants in the absence (―) or the presence of PhrF (—) and PhrC (····) as monitored by Sypro orange fluorescence. The Tm values from at least three independent experiments performed in duplicated are indicated.

Peptide anchoring should be independent of its sequence. Thus, it is primarily mediated by interactions with the peptide main chain. These interactions are mainly polar in nature and involve the side chain hydrogen bonds and salt bridges of Tyr66, Tyr152, Gln183, Arg223, Asn227, Gln263, and Asp338 with the PhrF backbone (Figure 2). Additionally, the conserved Arg at position 2 of the Phr peptides is anchored by a salt bridge with the side chain of a conserved Asp residue (Asp194 in RapF) from TPR3 (Figure 2). This side-chain side-chain interaction was expected to be preserved between Raps and Phrs as both positions were strictly conserved, except for the PhrG-RapG pair where conservative Arg to Lys (PhrG) and Asp to Glu (RapG) changes were observed (Figure 2B), changes that should maintain the salt bridge. Previous mutagenic assays have demonstrated the pivotal role of these residues in peptide recognition and binding [11],[19],[25]. The position occupied by Asn227 in RapF was strictly conserved in the RNPP family (Figures 1E and 2B) and its mutation to alanine in RapA yielded a protein with impaired capacity to bind the PhrA peptide, but with intact capacity to bind its target RR Spo0F [11]. Furthermore, peptide backbone recognition mediated by asparagine interactions has been reported to be a conserved feature of either prokaryotic or eukaryotic TPR domains [4],[11],[13]. To confirm these observations, RapF Asn227 to Ala mutant was generated (RapF^N227A^) and its PhrF binding capacity was checked by a native gel electrophoresis assay. The electrophoretic mobility of RapF is altered upon PhrF binding and the RapF-PhrF complex presents faster mobility than RapF alone when employing native gels (Figure 3A) [34]. In the case of RapF^N227A^, no shifted bands were observed after incubation with PhrF (Figure 3A). Since RapF and RapF^N227A^ were similarly capable of interacting with their target protein ComA (Figure 3A), it would seem that the protein folding and stability of RapF were not compromised by the mutation. Indeed, PhrF had no effect on the RapF^N227A^ binding to ComA (Figure 3A), thus supporting the key role of this conserved residue in Phr-Rap recognition and the separate location for the Phr and ComA sites. The anchor role revealed by the RapF-PhrF complex for the positions occupied by Asp194 and Gln263 has been previously reported for RapA and RapC. Mutations of the equivalent positions in RapA (Asp192 and His260, respectively) generated variants that were insensitive to the inhibitory activity of PhrA, but with the unaffected ability to promote Spo0F∼P dephosphorylation [19],[35]. Equivalent mutations in RapC (Asp195 and Pro263 to Asn and Leu, respectively) also disrupted peptide binding, thus confirming the critical role of these positions to establish interactions that allow a complex formation between Raps and their inhibitory peptide [25]. The anchor function for Asp194 in RapF was confirmed by generating an Ala mutant in this position (RapF^D194A^). Similarly to RapF^N227A^, the mutation of Asp194 disrupted the capacity of RapF to bind PhrF, but had no effect on the binding to ComA (Figure 3A), thus validating the PhrF-bound structure and the proposed anchor role for this residue.

**Figure 4:**
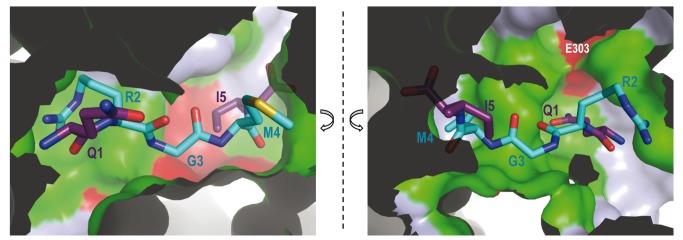
Peptide specificity. Rap proteins shown an exquisite specificity for their inhibitory peptides as is exemplified by the closely related PhrF (QRGMI) and PhrC (ERGMT) peptides and their targets RapF and RapC/RapB, respectively. The RapF peptide-binding site is represented in semi-transparent surface colored in green and red for conserved and variable residues, respectively, among RapF, RapB, and RapC. PhrF is shown in sticks rendering with carbon atoms colored in cyan for identical positions with PhrC and purple for variable. As in Figure 2, the active center is cut along the peptide axis and presented in two halves for an easier visualization.

Given their conservation, anchor residues should play a minor role in discriminating inhibitory peptides. The ability to distinguish different inhibitory peptides should be conferred by the variable residues among Rap proteins, which recognized particular side chains of each inhibitory peptide. Therefore, we propose that the residues described in the previous section (Figure 2B, residues highlighted in light blue), which interacted with the RapF side chains, would work as “specificity” residues. To confirm our hypothesis, and as a case in point, the RapF, RapB, and RapC sequences were compared and the positions that would mediate peptide specificity, guided by the RapF-PhrF structure, were mapped (Figures 2B and 4). RapC and RapB were inhibited by PhrC (ERGMT) [16], which differed from PhrF in terms of its first and last positions (QRGMI). Therefore, a search was made among the proposed specificity residues for those that interacted with positions 1 and 5 of PhrF, which were identical in RapB and RapC, but changed in RapF (Figures 2 and 4). A strong candidate residue was Glu303, which interacted with PhrF Gln1 by a hydrogen bond. This residue was substituted by a Lys in RapC and RapB, which could interact by a salt bridge with PhrC Glu1 (Figures 2, 4, and S5). Indeed, similar Lys–Glu (Rap-Phr) couples were observed for RapG and RapK, and also for their inhibitory peptides, PhrG and PhrK, respectively (Figure 2B). Furthermore, RapI, whose inhibitory peptide PhrI had an acidic (Asp) residue at position 1, also presented a Lys at this position (Figure 2B). The RapF mutant forms of Glu303 to Ala (RapF^E303A^) and Lys (RapF^E303K^) were constructed by the latter mutation emulating the residue observed in other Raps, and the peptide binding capacity of these forms was tested by native gel electrophoresis. As seen in Figure 3A, RapF^E303K^ was able to bind PhrC, but RapF^E303A^ did not display this ability; this scenario supports the prominent role of this position in peptide recognition. Both Glu303 mutants preserved their ability to bind ComA, but this ability was inhibited by PhrC with RapFE303K, but not with RapF^E303A^ (Figure 3A). This is in close agreement with the PhrC binding capacity of each RapF mutant and indicates that peptide binding and the induced conformational changes are similar to those produced by PhrF. Interestingly, PhrF interacted with both RapF Glu303 mutants (Figures 3A), suggesting that RapF^E303K^ Lys could maintain the interaction with PhrF Gln1, and in the case of RapF^E303A^, the absence of this contact did not suffice to abolish the RapF-PhrF interaction (at least not under our experimental conditions).

Thermal-shift assays were used to further confirm the peptide binding capacity of each mutant. PhrF induced a strong stabilization of RapF with an increment in the melting temperature of ∼20°C (from 49.5°C to 69.3°C), which supports peptide binding to the protein (Figure 3B). Unlike PhrF, non RapF partner peptides PhrA, PhrC, and PhrE presented identical melting transitions to those observed in the absence of peptide (Figure 3B and unpublished data), indicating the lack of RapF-peptide interaction. Similarly, the denaturation temperature for the RapF^N227A^ and RapF^D194A^ mutants was unaltered by PhrF or PhrC (Figure 3B), which was expected for the mutations that abolish peptide binding. The RapF^E303K^ mutant was stabilized by PhrF and PhrC (Figure 3B), which reveal this mutant's capacity to bind both peptides and the peptide specificity reduction by this mutation. The thermal denaturation of RapF^E303A^ also confirmed the previous electrophoretic results, showing that PhrF, but not PhrC, stabilized the protein (Figure 3B). As anticipated by their capacity to interact with ComA, this analysis also confirms that the mutations had no deleterious effect on protein folding or stability as all the proteins presented a similar melting point when the peptide was absent (Figure 3B).

### Quantitative Analysis of the RapF-PhrF Interaction

**Figure 5:**
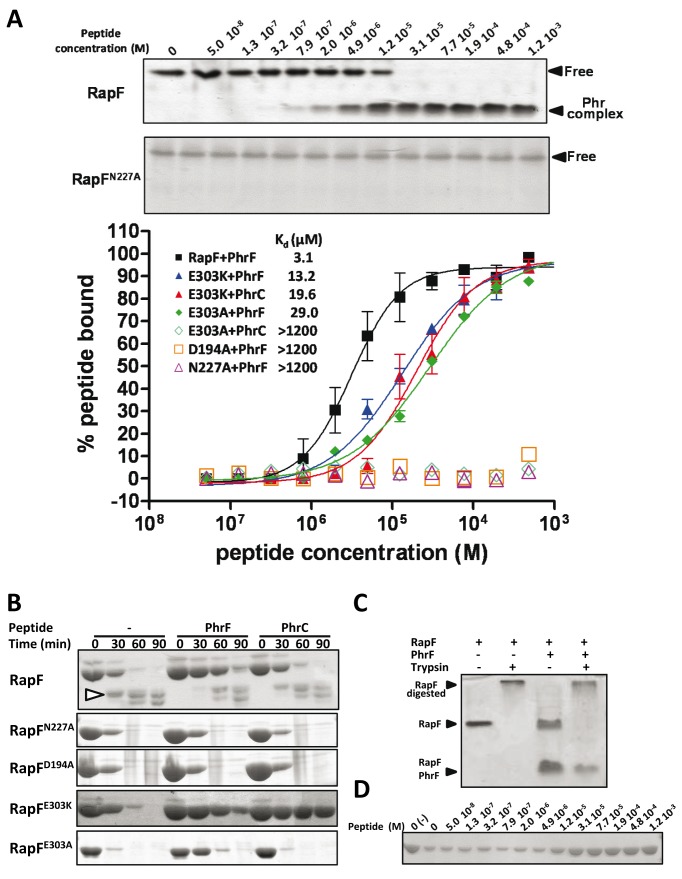
Quantification of Phr peptide binding to wild-type and mutational RapF variants and limited proteolysis analysis. (A) RapF or its mutational variants were incubated with increasing concentrations of PhrF and/or PhrC peptides (0–1.2 mM) and separated by native PAGE. The band corresponding to the Rap-Phr complex was quantified and represented versus peptide concentration to calculate the apparent constant affinities (K_d_). Representative PhrF titration experiments for Rap proteins with (RapF) or without (RapF^N227A^) capacity to bind the peptide are shown in the upper part of the figure. (B–D) Limited proteolysis of wild-type RapF and mutant forms. (B) Wild-type RapF or mutants were incubated with trypsin in the presence (1 mM) or absence of inhibitory peptides. The reaction were stopped at the indicated time points and analyzed by SDS-PAGE. The proteolytic fragment analyzed by Edman sequencing is indicated by a white arrowhead. (C) Native gel analysis of RapF trypsin digestion. RapF was digested for 60 min with trypsin in the presence or the absence of PhrF (1 mM) and analyzed by native gel electrophoresis. Notice that RapF-PhrF is selectively protected against the trypsin digestion. (D) Trypsin protection is peptide-concentration dependent. RapF was incubated with increasing concentrations of PhrF and subjected to limited proteolysis with trypsin for 60 min and analyzed by SDS-PAGE. Line labeled with (-) corresponds to a control without trypsin.

To quantify the effect on the Phr peptide binding of the RapF mutations described in the previous section, we calculated the apparent binding affinity (*K*_d_) for Phr peptides by gel shift assays. Titration of RapF with PhrF showed that less than 30% of RapF was present in the free form at a concentration of 12 µM PhrF (Figure 5A, upper panel), and a *K*_d_ value of 3.1 µM for the PhrF peptide was calculated. In contrast, the PhrF-protein complex was not observed for the RapF^N227A^ or RapF^D194A^ mutants, even if the peptide was present at concentrations as high as 1.2 mM (Figure 5A, upper panel). Therefore, these point mutations abolished the peptide-binding capacity of RapF supporting their anchor role proposed herein. A similar analysis of the RapF^E303K^ and RapF^E303A^ mutants confirms the key function for the residue at position 303 in the peptide selection since its mutation to Lys, but not to Ala, conferred RapF the previously absent capacity of PhrC binding (Figures 3 and 5A). Quantification of this interaction showed a K_d_ of 19.6 µM for the binding of PhrC to RapF^E303K^, which was only ∼6 times lower than that calculated for RapF-PhrF (Figure 5A). This finding indicates that the Rap-Phr interaction was the result of a complex set of interactions, many of which were common for PhrC and PhrF, but some positions contributed more in selecting the peptide by recognizing the distinctive peptide positions. The quantification of PhrF binding to the Glu303 mutants supports the “specificity” character of this position since its change to a residue, which eliminated the interaction with the peptide as Ala, only reduced (∼10 times; *K*_d_ 29 µM), but did not abolish, the binding capacity of RapF; this was the case of the equivalent substitutions at anchor positions Asp277 and Asp194 (Figure 5A). In addition, the substitution of Glu303 by Lys, a residue that was still able to maintain the interaction with PhrF Gln1, only diminished the binding affinity (*K*_d_ 13.2 µM) for this peptide by ∼4 times in relation to the wild-type protein (Figure 5A).

### RapF Conformational Changes Induced by PhrF Binding

**Figure 6:**
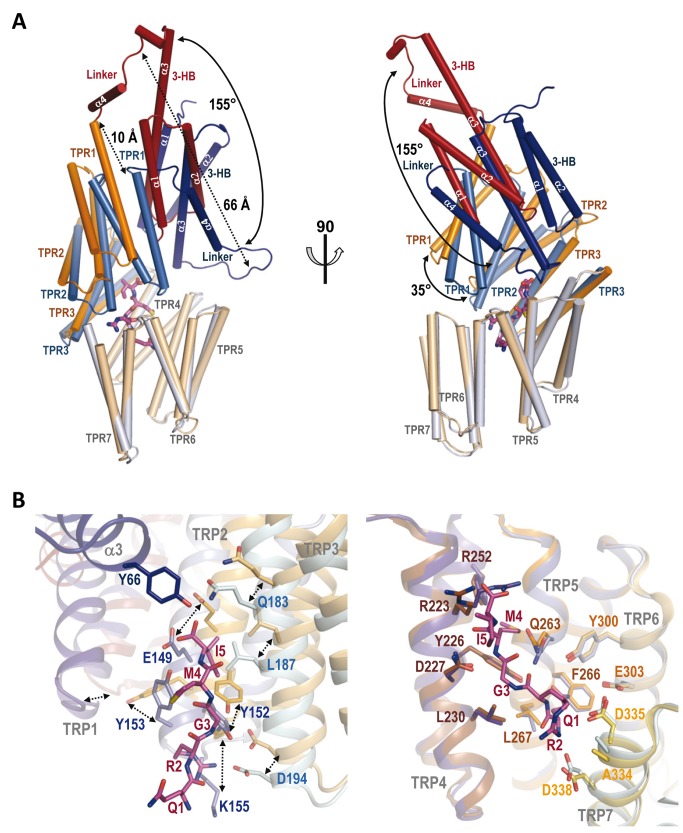
Conformational changes in RapF upon PhrF binding. (A) Superimposition of RapF structures from the PhrF (blue hues) and ComA (orange hues) [6] complexes. The view shows two cartoon diagrams (90° rotation along the vertical axis). Superimposition reveals a huge (155°) angular movement of approximation to the TPR domain of the N-terminal portion (darker hues) induced by PhrF (in sticks rendering with carbon atoms colored in pink) binding. TPR1–TPR3 motifs (bright hues) are displaced towards the TPR4–TPR7 motifs (tint hues) that present an almost fixed disposition in both structures. Helices are shown as cylinders and labeled for the N-terminal domain. TPR motifs are labeled in the TPR domain. (B) Detailed view of the conformational changes induced in TPR1–TPR3 (left) and TPR4–TPR7 (right) motifs of RapF upon PhrF binding. TPR motifs are represented in ribbon, labeled, and colored in blue and orange hues for RapF in complex with PhrF and ComA, respectively. Interacting amino acids are shown in sticks, labeled, and colored with carbons in the same color that the corresponding TPR motif. Dashed lines indicate the displacements. PhrF is shown as a stick model with carbons colored in pink.

A comparative analysis of the RapF structures revealed major conformational changes upon peptide binding. PhrF accommodation induced TPR domain constriction, which is mediated by the displacement of some TPR segments. The conformational changes proved even more drastic in the 3-helix bundle domain, which underwent a rotation of around 155° in relation to the position observed in the peptide-free RapF structures (Figures 6A and S4). The 3-helix bundle location observed in crystal was genuinely induced by the peptide and was not promoted by lattice contacts given that this domain was solvent-exposed (the solvent content of the RapF-PhrF crystals was 75%) and participated minimally in crystal packing. Since PhrF was recognized by the TPR domain, we compared the underlying structural parameters of the superhelical coil arrangement of this domain in the RapF structures. Our analysis shows that the superhelical pitch was reduced by ∼7 Å in the PhrF complex, approaching the TPR1–TPR3 motifs to the remaining TPR segments (Figure 6). This superhelix pitch reduction was reflected in the apical portion of the TPR channel, where the TPR domain width was reduced by more than 10 Å in the PhrF complex (from 23 Å to 11 Å) by the channel narrowing to about 2,500 Å^3^ (Figures 6A and S4). However, the exposed channel surface increased by about 350 Å^2^ (calculated by CASTp) since an additional area was provided by the 3-helix bundle, which the peptide-induced displacement placed in the channel (Figures 6 and S4).

It was possible to dissect the rearrangement of the global domain induced by the peptide into local movements. In this way, PhrF binding was seen to have a strong effect on the relocation of the TPR1, TPR2, and TPR3 (residues 98–215) segments, although TPR4–TPR7 (residues 216–368) underwent minor movements (Figure 6). The superimposition of the complete TPR domain (residues 98–368) of both RapF structures yielded a large root mean square deviation (RMSD) of 3.9 Å. However, the last four TPR segments (TPR4–TPR7) superimposed extremely well (RMSD of 0.8 Å for 152 residues), unlike the largest differences (RMSD of 2.6 Å for 117 residues) observed for the first three TPR segments (Figure 6). Therefore, the TPR4–TPR7 segments seemed to be a fix preformed bed where the peptide was placed. Indeed the side chains of the residues of these TPRs, which interacted with the peptide, underwent minor movements (>0.5 Å) and maintained their conformation in both RapF structures, except for Arg223, which presented a different rotamer (Figure 6B). These interactions mainly include those termed herein as anchoring residues (except Tyr152 from TPR2), which recognized the peptide main chain, thus supporting that the TPR4–TPR7 fragment of the Rap proteins preformed the Phr binding site. In addition, those residues interacting with the side chains at peptide positions 1 and 4 also presented a fixed disposition, except for the previously indicated Arg223 in both RapF structures, and could be considered part of the defined peptide bed. Conversely, the side chains at positions 2 and 5 of the peptide faced the TPR segments (TPR1–TPR3), which underwent major movements after PhrF binding (Figure 6B). To interact with PhrF, these TPR segments were enforced to displace toward the peptide binding bed in a rotation movement of about 15 degrees (calculated by DynDom [36]). RapF TPR3 was displaced by ∼3 Å because of the interactions of residues Gln183, Leu187, and Asp194 with peptide residues Arg2 and Ile5 (Figure 6B). Displacement was more marked in TPR2, which moved about 5 Å by positioning the side chains of Phe145, Glu149, Tyr152, and Tyr153 to contact distance of Arg2 and Ile5 (Figure 6B). TPR2 and TPR3 approached the peptide through a rigid body movement, which included the side chains of the peptide-contacting residues, except for Tyr153 from TPR2, whose side chain acquired a different rotamer to interact with PhrF Arg2 and Ile5 (Figure 6B). Unexpectedly, TPR1, the unique TPR segment that did not interact with peptide TPR1, underwent the longest displacement in the TPR domain dragged by its tight interaction with TPR2. With a rotation of about 35 degrees (calculated by DynDom) in relation to TPR2, and with a displacement of around 10 Å (Figure 6), TPR1 relocated over the TPR domain and closed its internal channel. The new position of TPR1 was stabilized by the interactions between the residues from the TPR1 α1–α2 loop (110–-118) with the residues from the loops connecting the TPR5–TPR6 and TPR6–TPR7 motifs (294–299 and 328-–334). As the TPR1 residues (Arg115, Tyr117, and Leu118) involved in these interactions mediated the contacts with the residues from the C-terminal tail (371–374) in the RapF-ComA structure, the movement of TPR1 toward the peptide binding pocket would be responsible for the C-terminal tail disorder in the RapF-PhrF structure.

**Figure 7:**
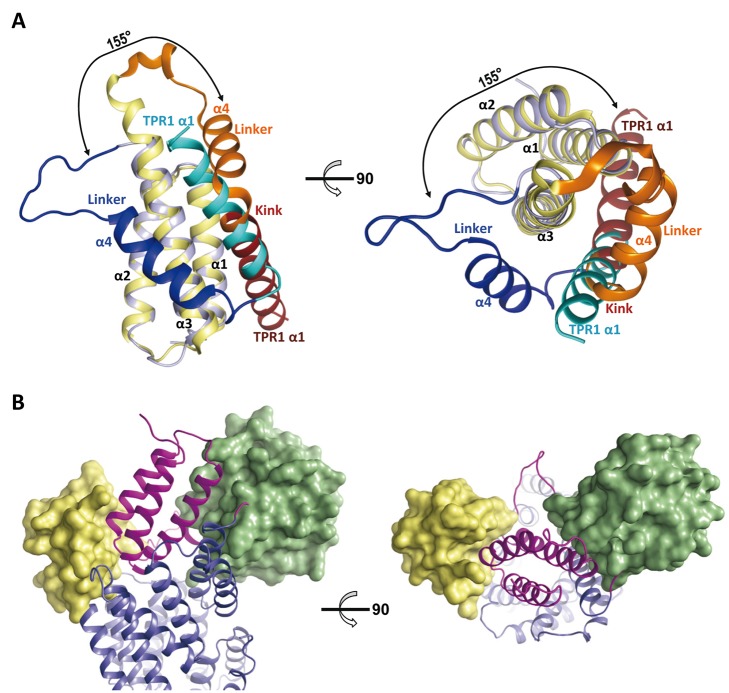
PhrF induced conformational changes disrupt response regulator binding sites. (A) Side (left) and top view (right) of the superimposition of RapF 3-helix bundle from the PhrF (blue hues) and the ComA (yellow hues) complexes shows that this domain rotates ∼155° as a rigid body using the linker region (dark blue and orange for PhrF and ComA complexes, respectively) as a hinge. As a result of the movement α1 helices of TPR1 motif (cyan and dark red for PhrF and ComA complexes, respectively) superimpose but with inverted orientation, extending from three to five helices the helix bundle in the case of RapF-PhrF complex. (B) RR binding to Rap is impaired in the Phr-induced conformation. Spo0F and DNA-binding domain of ComA (green and yellow surfaces, respectively) were placed in the RapF-PhrF structure by aligning the 3-helix bundle of RapF-Spo0F (3Q15) [6] and RapF-ComA (3ULQ) [7] with RapF-PhrF structure. RapF-PhrF is represented in ribbon and colored the N-terminal portion in magenta and the TPR domain in blue, view from the side (left) and the top (right).

The pronounced movement of the TPR1 segment triggered by PhrF is more notorious in the 3-helix bundle. As TPR1 connects with the 3-helix by the linker region, it is a plausible line of reasoning that TPR1 movements could be propagated to the 3-helix bundle by a lever movement of the linker region, which is involved in ComA recognition, and comprises a long flexible loop and the α4 helix (Figures 1 and 7A). This short helix (81–91) is separated from the TPR1 α1 helix by a 3-residues kink (92–94) in the RapF-ComA structure to form 90 degrees between both structural elements (Figure 7A). However, the RapF-PhrF structure revealed that the α4 helix ran almost in parallel to the TPR1 α1 helix (Figure 7A) after a ∼155-degree rotation forced by the movement of the TPR1 α1 helix. The concomitant rotational movement of the 3-helix bundle brought this domain over the channel of the TPR domain (Figures 6A and S4) in a position that became stabilized through several contacts with the TPR domain, including the interaction of Tyr66 with the peptide. The rotational movement was accomplished in a rigid body manner since the superimposition of this domain in both RapF complexes presented an extremely good fitting (0.7 Å RMSD) (Figure 7A). In the new disposition, the α3 helix of the 3-helix bundle was located between linker α4 and the TPR1 α1 helices and, in this way, the original bundle extended from three to five helices (Figure 7A). Although the 3-helix bundle presented an almost inverted disposition in each complex, the interface between the 3-helix bundle and the TPR1 α1 helix involved a similar set of residues, but with swapping interactions among them. A sequence alignment of all the Raps in *B. subtilis* shows that the residues implicated in these contacts were strictly or highly conserved (one or two changes of the 11 Rap sequences), which strongly suggests that the contact network and the orchestrated movements enabled by them, described herein for RapF, could represent a general mechanism in the Rap family

### Molecular Mechanism of RapF Inhibition by PhrF

We have shown that PhrF binding induces drastic movements of the RapF 3-helix bundle. But, how do these conformational changes promote RapF inhibition? It has been suggested that Phr peptides could compete with RRs to bind to a common site [25], but recent genetic and biochemical experiments support that Phr peptides and target RRs bind at distinct sites of the Rap proteins [11]. The structures reported here confirm that the ComA and PhrF binding sites are independent and corroborate that PhrF mediates action allosterically by the conformational changes described in the previous section. Residues from the 3-helix bundle and the linker region accounted for the binding of ComA (Figure 1D and 1E) [6], unlike PhrF which was recognized by the TPR domain. Surprisingly, the movement of the 3-helix bundle induced by PhrF did not completely occlude the ComA binding site provided by this domain (Figure 7B). Nonetheless, the 3-helix bundle relocation induced the unfolding of the two final helix turns of the α3 helix (residues 67–73), which went on to form part of the linker region (Figure 7A). In this way, the linker region was six residues longer in RapF-PhrF and had a reverse disposition (rotation of about 155 degrees) due to the rotational movement of 3-helix bundle (Figure 7A). Thus, the RapF residues interacting with ComA from the last turns of the α3 helix and the linker region had a completely different disposition (Glu68 and Glu71) or faced the TPR domain (Glu78, Arg80, and Leu81) in the RapF-PhrF structure. Since these RapF residues provided ∼40% of the interaction surface with ComA, the binding of ComA to RapF-PhrF would be largely compromised. Actually, the in vitro and in vivo assays showing that the single mutation of one of these residues, Gln78, to alanine significantly impaired RapF-ComA binding [6] supports this statement. Although the remaining ComA binding site in the 3-helix bundle was almost conserved, binding of ComA would be sterically prevented by the relative disposition of the 3-helix and the TPR domains triggered by PhrF binding (Figure 7B). All together, the partial disruption and sterical obstruction of the ComA binding site explain PhrF-induced RapF inhibition.

In order to experimentally correlate the peptide binding with the conformational changes observed in the 3-D structure, we performed a limited proteolysis study of RapF and its mutational variants in the absence and presence of PhrF and PhrC peptides. Tryptic proteolysis of RapF gives a major fragment of 35 KDa (Figure 5B), whose N-terminal sequencing yielded the “LSDLLLE” sequence, corresponding to a cut of trypsin in Arg80 that generated a C-terminal RapF fragment (Leu81-Val381) with a theoretical molecular weight of 35.617 Da (Figures 1E). Arg80 was located in a loop of the linker region, participated in the ComA binding and was exposed to the solvent in the RapF free from (Figures 1E and S6). In contrast, the PhrF-induced displacement of the N-terminal portion of RapF reduced Arg80 exposition, protecting the protein from tryptic digestion (Figure S6). The assay was validated using non inhibitory RapF peptides, such as PhrC (Figure 5B), PhrA, and PhrE (unpublished data), as controls, but they did not protect against trypsin attack. In addition, the analysis by native PAGE showed that trypsin selectively cut the RapF band corresponding to the peptide-free protein, but not the peptide-bounded protein (Figure 5C), supporting the connection between peptide binding and the conformational changes in the N-terminal portion that guards RapF against digestion. Accordingly, the protective effect of PhrF was lost in the RapF^N277A^ and RapF^D194A^ mutants, which were unable to bind the peptide (Figure 5B). The RapF^E303K^ mutant, with affinity to PhrF and PhrC, was protected by both peptides, indicating a similar conformational change induced by PhrF and PhrC (Figure 5B). Finally, the tritation of RapF protection displayed a similar PhrF concentration-dependent behavior to RapF peptide-binding affinity (Figure 5A and 5D), confirming the direct relationship between peptide binding and 3-helix bundle displacement as inferred from its resistance to be hydrolyzed by trypsin. By taking our structural and functional data together, both lines of evidence demonstrate that peptide binding in the TPR domain induced a conformational change that propagated to the N-terminal portion of the protein, which adopted a completely different disposition in the RapF-PhrF complex and was impaired by ComA binding.

## Discussion

Raps are regulatory proteins that modulate the signaling activity of two component systems in two completely different ways: dephosphorylation or blocking the DNA-binding activity of their RR target. Rap proteins are themselves in turn inhibited by specific Phr peptides, adding an extra level of complexity to this regulatory mechanism. Despite the extensive sequence conservation shared by Rap family members, Rap-Phr couples are highly specific. Recent structural studies have shown the molecular architecture of these proteins and the mechanism of RR recognition and inhibition, which involve the Rap N-terminal portion, independently of the inhibitory mechanism followed by the Rap protein [6],[7]. Here, we present structural and functional data to elucidate how Rap proteins are inhibited by Phr peptides at the molecular level. We show how PhrF blocks the interaction of RapF with its target RR by an allosteric mechanism. By binding to the TPR domain, this peptide constricts this domain that propagates to the 3-helix bundle domain, which, in turn, is relocated by a pronounced rotational movement. The new disposition of the N-terminal portion partially disrupts the binding site of the RR, whose binding to the Rap protein is also sterically impaired by the relative disposition of both the 3-helix bundle and the TPR domains.

Our structural data confirm the proposed ligand recognition and regulatory function for the TPR domains in the RNPP proteins [4]. As previously demonstrated for other RNPP family members, PlcR and PrgX, the regulatory peptide binds in an extended conformation to the concave side of the TPR superhelix [4],[9],[12]. The conserved asparagine in the RNPP family (Asn227 in RapF) interacts with the PhrF backbone and supports the key role of this residue in peptide accommodation. The impaired capacity of RapF^N227A^ to bind PhrF confirms its proposed structural function in peptide fixation, as previously suggested by functional assays in RapA and RapC [11]. Six additional RapF residues (Tyr66, Tyr152, Gln183, Arg223, Gln263, and Asp338) mediate interactions with the PhrF backbone. The medium (Arg223, Gln263, and Asp338) or high conservation (Tyr66, Tyr152, and Gln183) feature of these residues suggests a predominant participation of these positions in the peptide binding for all Raps. Indeed, the mutation to alanine of Tyr224 or His260 in RapA (corresponding to RapF Tyr226 and Gln263, respectively) completely abolishes the capacity to bind PhrA, its inhibitor peptide, but has no effect on enzymatic activity [11]. Therefore, we named the residues in these positions “anchor” since they should ensure peptide binding in the proper disposition in the TPR domain of different Raps, irrespectively of the peptide sequence. Additionally, the position corresponding to the conserved Asp194 in RapF should also form part of the anchoring machinery but, in this case, it should interact by a side-chain side-chain salt-bridge with the conserved positive charge at position 2 of the Phr peptides. The mutation of Asp194 in RapF described herein or the corresponding aspartic residues of RapA and RapC can result in proteins that are insensitive to inhibitory peptides, but which conserve activity toward their target RR [11], thus supporting the functional role of this residue. In contrast, peptide discrimination should be accomplished by a set of characteristic residues of each Rap protein to ensure Rap-peptide specificity. The RapF-PhrF structure reveals that up to 15 residues might be implicated in peptide discrimination, thus we called them “specificity” residues. Given the partial sequence conservation of inhibitory peptides and the fact that the same peptide can inhibit several Raps (Figure 2B), some of these positions are partially conserved among different Raps. These residues are spread along six of the seven TPR motifs, which is in agreement with the extending peptide disposition. RapF peptide selectivity was relaxed by mutating only one of these residues (Glu303), which validates the functionality of the proposed recognition residues. In addition, the participation of these positions in peptide binding has been confirmed in RapA by the mutation of two of these residues (corresponding to RapF Tyr226 and Leu230), which generate functional RapA variants, although they are completely insensitive to the PhrA peptide [11]. However a more detailed study, guided by the structural data provided herein, is required to evaluate each residue's particular contribution to the peptide discrimination process.

The RapF-phrF structure has revealed that peptide binding to the center of the concave side of the TPR motif triggers an allosteric mechanism that rearranges the N-terminal RR binding portion. The drastic conformational changes induced by the peptide observed in RapF can be anticipated by the extremely different behavior of RapF and the RapF-PhrF complex in native-PAGE, gel filtration, or analytical ultracentrifugation [6]. The new disposition of the 3-helix bundle domain hides and disorders the RR binding site, and inhibits Rap protein activity. The mechanism described herein resembles that reported for other RNPP family members, such as PlcR and PrgX. For these proteins, the binding of the cognate peptide also induces a closure of the TPR domain, with the consequent conformational change in the N-terminal domain. However, some major differences are seen between these two RNPP members and RapF. First, it has been reported that RapF and its complex with ComA are monomers in solution [6], thus PhrF binding does not induce any change in the oligomeric state of RapF. In contrast, signaling peptides have a major impact on the quaternary state of PlcR and PrgX by inducing oligomerization (from dimer to tetramer or dodecamer) in the former and de-polymerization (form tetramer to dimer) in the latter [4],[9]. Second, the rearrangement experience for the N-terminal effector domain induced for the peptide in PrgX and PlcR is moderate if compared with the ∼155° rotation undergone in the case of RapF. Finally, the signaling peptide induces conformational changes in RapF, which partially disorganize the RR binding site, but no major alterations in the DNA binding domain are induced by the signaling peptide in PrgX [9],[12]. In short, it seems that the molecular mechanisms of effector peptides recognition and allosteric regulation are conserved in the RNPP family, but that the final effect induced in each family member is apparently quite different.

The structural comparison of RapF-ComA and RapH-Spo0F shows that both proteins are almost structurally identical, but that the respective RR binding sites are placed differently. Based on the structural similarity between both Rap proteins, including the residues that work as a hinge in the conformation change induced by the inhibitory peptide, it is worth speculating that RapH can be inhibited by a similar allosteric mechanism to that described herein for RapF. Mapping the RapH residues that conform the Spo0F binding site onto the RapF-PhrF structure reveals that RR binding may be sterically precluded if RapH adopts a similar disposition to RapF-PhrF induced by PhrH (Figure 7B). Therefore, we anticipate that the molecular mechanism described herein could be general for Rap proteins as the effector peptides mediate their inhibitory activity allosterically by promoting a N-terminal portion conformational change that prevents the RR–Rap interaction. Additional structures of Rap proteins in complex with their inhibitory peptides are crucial to generalize the mechanism described herein.

In conclusion, our results elucidate the molecular mechanism used by effector peptides to inhibit Rap proteins, and provide insights into the molecular basis of peptide discrimination and binding. These data also generate valuable information for the rational design of tools to study signaling mediated by these systems. In this way, it is possible to engineer Rap proteins with wild-type activity that are completely insensitive to their inhibitory peptides or Rap proteins with relaxed peptide selectivity.

## Materials and Methods

### Protein Expression and Purification

*RapF* was amplified from *B. subtilis* genomic DNA using oligonucleotide primers RapF5′BamHI and RapF3′NcoI (Table S2). The PCR product was purified by agarose electrophoresis, digested with BanHI and NcoI restriction enzymes and cloned into the BamHI–NcoI site of pPROEX-HTa plasmid (Invitrogen). Similarly, full-length ComA was cloned onto the NcoI–HindIII site of the pPROEX-HTa vector using oligonucleotide primers ComA5′NcoI and ComA3′HindIII (Table S2). The resulting plasmids, pPROEX-RapF and pPROEX-ComA, respectively, fused to RapF and ComA, an N-terminal tail consisting of six histidines (6×His), followed by a TEV protease recognition sequence. RapF and ComA were purified following an identical protocol. pProEX-RapF or pProeX-ComA was transformed into *Escherichia coli* expression strain BL21 RIL (Novagen). A single colony for each transformation was grown overnight at 37°C in 100 ml of LB medium supplemented with 100 µg/ml of ampicillin and 33 µg/ml of chloramphenicol. This culture was used to inoculate 3 l of LB medium containing ampicillin (100 µg/ml) and chloramphenicol (33 µg/ml), and was grown at 37°C until cells reached an optical density at 600 nm (OD_600_) of 0.4; the temperature was set at 30°C. After 30 min, the expression of RapF was induced with isopropyl-ß-D-thiogalactopyranoside (IPTG) at a final concentration of 0.4 mM and the culture was incubated for 4 h at 30°C. Cells were then harvested by centrifugation and the pellet was resuspended in lysis buffer (20 mM TrisHCl [pH 8], 200 mM NaCl, 10 mM imidazole) and lysed by sonication on ice. Cell debris was removed by centrifugation at 10,000 g for 1 h. The supernatant was filtered through a 0.45 µm syringe filter and loaded onto a 5 ml HisTrap FF (GE Healthcare) and rinsed with lysis buffer until the baseline stabilized. The 6×His proteins were eluted in lysis buffer supplemented with 200 mM imidazol. The fractions containing the purest protein evaluated by SDS-PAGE stained with Coomassie blue were pooled and digested with TEV protease (50∶1 molar ratio protein∶TEV) and dialyzed against a 500 times volume of dialysis buffer (20 mM TrisHCl [pH 8], 200 mM NaCl). The sample was concentrated by ultrafiltration through a 30-KDa microfilter (Millipore) and was loaded in a Hi-Load Superdex 200 16/60 (GE Healthcare) gel filtration column equilibrated with dialysis buffer. The purest fractions judged by SDS-PAGE were pooled, concentrated by ultrafiltration and stored at −80°C. Typical yields were 20–30 mg recombinant protein/l of culture medium. Selenomethionine-substitute (SeMet) RapF was obtained by growing the *E. coli* strain in M9 minimal medium supplemented with 0.001% biotin, selenomethionine (50 mg/ml) as well as amino acids inhibiting methionine synthesis (isoleucine, leucine, and valine at 50 mg/ml; lysine, phenylalanine, and threonine at 100 mg/ml). Purification protocol for SeMet protein was identical to the native protein.

### Site-Directed Mutagenesis

Site-directed mutagenesis was performed with the QuikChange mutagenesis kit (Stratagene) according to the manufacturer's instructions. The pPROEX-RapF plasmid carrying the *rapF* wild-type gene was used as a template and mutations were introduced utilizing the oligonucleotides listed in Table S2. Methylated parental DNA was then degraded by adding 10 units of DpnI (Fermentas) to each PCR reaction and by incubating for 2 h at 37°C, followed by the transformation of *E. coli* strain DH5-α. Positive *rapF* mutant plasmids were screening by PCR amplification and sequencing. Mutant proteins were expressed and purified as native RapF.

### Protein Crystallization and Data Collection

RapF-PhrF crystals were grown by the sitting-drop vapor-diffusion method at 21°C by mixing an equal volume of RapF-PhrF (RapF 10 mg/ml, PhrF 1 mM purchased from Genescript) and reservoir (1 M Ammonium Sulfate, 17% Glycerol, 0.1M Tris [pH 8.5]). Crystals grew in 2–3 d. For phasing, crystals were soaked for 24 h in reservoir solution, which also contained 0.01 mM terpyridine platinum. Crystals were directly flash-frozen in liquid nitrogen. A single-wavelength dataset to a maximum resolution of 3.8 Å from the Pt derivative crystal was collected at ESRF (Grenoble beamline BM14). A native dataset diffracting to 3.1 Å was collected on BL-13 Xaloc beamline (ALBA-Barcelona) (Table S1).

Native and SeMet crystals of RapF in the free form were grown by the sitting-drop vapor-diffusion method at 21°C by mixing an equal volume of RapF at 10 mg/ml with reservoir solution (1.2 M ammonium sulfate, 0.5 M lithium chloride). Crystals were cryoprotected in mother solution with 30% sucrose and flash-frozen in liquid nitrogen. A three wavelengths (peak 0.979 Å, inflection 0.9791 Å, and remote at 0.9074 Å) MAD experiment to a maximum resolution of 3.4 Å from the SeMet crystal was collected at ESRF, beamline ID14-1. A native dataset to 2.25 Å was collected at ESRF, beamline ID 14-4.

### Phase Determination and Refinement

The RapF-PhrF structure was determined by SIRAS using the data from the native and Pt-derivative RapF-PhrF crystals, which were isomorphous. Autorickshaw [37] was used to locate heavy atom, calculate phases, extend phases, and to build the initial model at a resolution of 3.4 Å. The final model was generated by interactive cycles of manual model building with Coot [38] and computational refinement with Refmac [39]. Free RapF crystal structure was determined by MAD using the data form the native and SeMet crystals, which were isomorphous. PHENIX [40] was used to locate selenium atoms, calculate and extend phases, and to build the initial model at 2.4 Å resolution. The final model at 2.25 Å resolution was generated by interactive cycles of manual model building with Coot [38] and computational refinement with PHENIX [40]. For both structures, solvent molecules were added to the final model using the find waters application of Coot [38]. Refinement statistics and models composition are shown in Table S1. Stereochemical properties were assessed by Molprobity [41] and Procheck [42]. Superimpositions were calculated using Lsqkab implemented in the CCP4 suite [43]. Surface accessibility was calculated using the CASTp software [32] and domain movements were calculated by DynDom [36]. Coordinates are deposited in the RSCB Data Bank under 4I9C and 4I9E for RapF-PhrF complex and RapF free structures, respectively.

### Native Gel Protein Analysis

The formation of complexes was analyzed by native polyacrylamide gel electrophoresis (Native-PAGE), similarly to that described by Salinas et al. [44]. Briefly, mixtures of RapF (WT and mutants), ComA (10 µM of each protein), or Phr peptides (0.5 mM), as indicated, were incubated in buffer A (200 mM NaCl, 50 mM Tris-HCl [pH 8.0]) at room temperature for 30 min, separated by Native-PAGE and visualized by Coomassie blue staining. The peptide binding constants for RapF, RapF^D194A^, RapF^N227A^, RapF^E303K^, and RapF^E303A^ were calculated following the same approach. Reaction mixtures containing 20 µg of protein and peptide concentrations ranging from 50 nM to 1.2 mM in 20 µl of buffer A were incubated for 15 min at room temperature. Reactions were separated in 8% acrylamide native gel (100 V, 4°C, 2 h) and visualized by Coomassie blue staining. Free protein was quantified using the Multigauge V2.1 software (Fujifilm) and the fraction of bound protein was estimated by subtracting free protein from the input protein. Data were plotted as a fraction of the Phr bound protein versus the Phr concentration, and were analyzed by the Graphpad prism software. The midpoint of the transition was taken as the apparent K_d_. To calculate the K_d_ values, at least three independent experiments were analyzed.

### Limited Proteolysis of Rap Proteins

Wild-type RapF and mutant variants (RapF^E303K^, RapF^E303A^, RapF^N227A^, and RapF^D194A^) at a concentration of 1.5 mg/ml were pre-incubated in lysis buffer (10 mM Tris-HCl [pH 8], 0.1 mM EDTA, 1 mM β-mercaptoethanol, 100 mM NaCl, 5% glycerol, and 5 mM CaCl_2_) in the presence (1 mM) or absence of inhibitory peptides at room temperature for 30 min. The protein was subjected to proteolysis by trypsin (1∶10,000) at 37°C and aliquots of the reaction mixture were taken after 0, 10, 30, and 90 min of incubation, mixed with SDS–PAGE loading buffer and immediately boiled to stop the enzyme reaction. Samples were then separated on 12% acrylamide SDS–PAGE and stained with Coomassie brilliant blue. For the peptide titration experiments, RapF was incubated in lysis buffer with increasing concentrations of PhrF at room temperature for 30 min and subjected to limited proteolysis with trypsin(1∶10,000) at 37°C for 60 min. The reactions were stopped SDS–PAGE loading buffer and analyzed by SDS–PAGE.

### Thermal-Shift Peptide Binding Assays

The thermal-shift assay was conducted in a 7500 Fast Real-time PCR System (Applied Biosystems). Final melt conditions were 20 mM Tris-HCl (pH 8), 100 mM NaCl, 20 µM RapF, and 5× SYPRO Orange (Sigma-Aldrich). The final concentration of the inhibitory peptides was 1 mM when they were present. The assays were carried out at a final volume of 20 µl in 96-well PCR plates. Samples were heated at 1°C/min. Fluorescent intensity was plotted *versus* temperature and integrated with the GraphPad Prism 4 software using a Boltzmann model to calculate melting temperatures.

## Supporting Information

Figure S1 **Electron density map of RapF-PhrF structure.** View of the 2F_o_-F_c_ electron density map at 3.1 Å, calculated using phases from the refined model and contoured at 1 σ. The view is center in the PhrF peptide (carbons in magenta) and also shows some interacting residues from RapF (carbons in yellow).
(TIF)

Figure S2 **RapF free and RapF-ComA structures present similar conformation.** Superimposed RapF structures in the presence (3ULQ; yellow and orange for RapF and ComA, respectively) and in the absence (light blue) of ComA binding domain. Both structures present an extremely similar conformation with local structural variability around the ComA binding site.
(TIF)

Figure S3 **Structural comparison of RNPP family members.** The structures of *B. subtilis* RapF-PhrF, *B. cereus* PlcR-PapR (2QFC) [4], and *Enterococcus faecalis* PrgX-cCF10 (2AXU) [9] are shown in the same view and represented in ribbon with the N-terminal effector domains colored in white and the TPR domains in rainbow. The corresponding peptides are shown in sticks rendering with carbon atoms in pink.
(TIF)

Figure S4 **TPR channel is constricted by peptide binding.** Three lateral views of RapF-ComA (upper panel) and RapF-PhrF (lower panel) structures in surface representation with N-terminal portion and TPR domain colored in yellow and blue, respectively. TPR channel surface, as is calculated by CASTp software [32], is colored in magenta.
(TIF)

Figure S5 **Rap peptide specificity is conferred by particular residues.** Model of the close related RapC-PhrC (left) complex based in the RapF-PhrF (right) structure illustrates the structural bases of peptide specificity. Hydrogen bond between RapF Glu303 and PhrF Gln1 (showed as black dashed line) is substituted by a salt-bridged between the Lys in positions 303 of RapC and the Glu in position 1 of PhrC (black dashed line).
(TIF)

Figure S6 **Arg80 localization in RapF structures.** The position of Arg80 (sticks with carbons colored in pink) is shown in the structures of free RapF and in complex with ComA and PhrF. The 3-helix bundle, linker region and TPR domain are colored in yellow, red, and light blue, respectively. ComA is colored in green and the ComA residues interacting with Arg80 are shown in sticks with carbons in green. Arg80-ComA interactions are highlighted with dashed black lines.
(TIF)

Table S1 **Data collection and refinement statistics.**
(DOCX)

Table S2 **Oligonucleotides.**
(DOCX)

## Abbreviations

RR: response regulator
TPR: tetratricopeptide repeat
