# Supplementary figures and images for: Structural Basis of Rap Phosphatase Inhibition by Phr Peptides
Source: PLoS Biol. 2013 Mar 19;11(3):e1001511. doi: 10.1371/journal.pbio.1001511 (PMC3601957; doi:10.1371/journal.pbio.1001511)

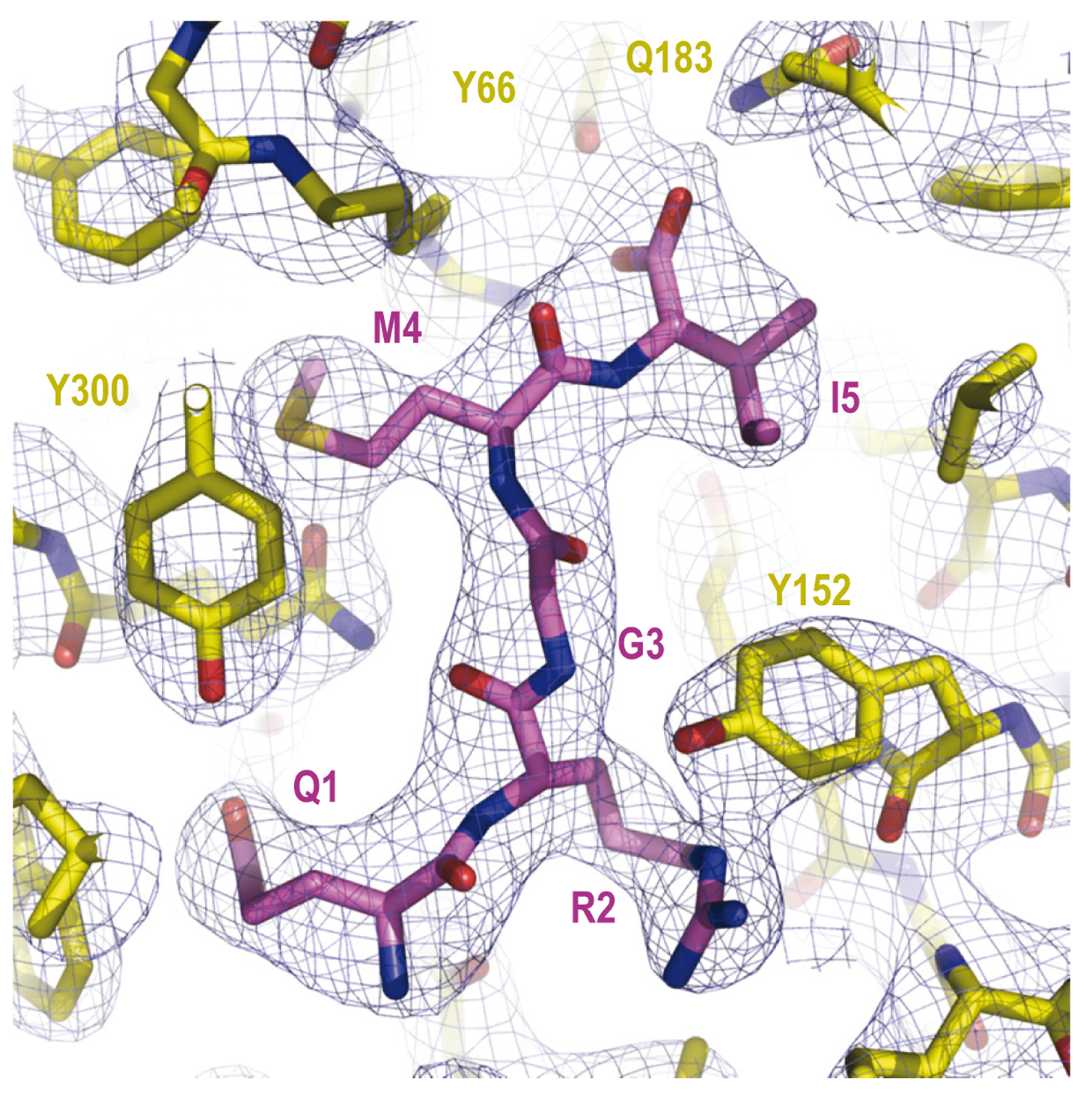

Supplement: Figure S1 — Electron density map of RapF-PhrF structure. View of the 2Fo-Fc electron density map at 3.1 Å, calculated using phases from the refined model and contoured at 1 σ. The view is center in the PhrF peptide (carbons in magenta) and also shows some interacting residues from RapF (carbons in yellow). (TIF) [file pbio.1001511.s001.tif]

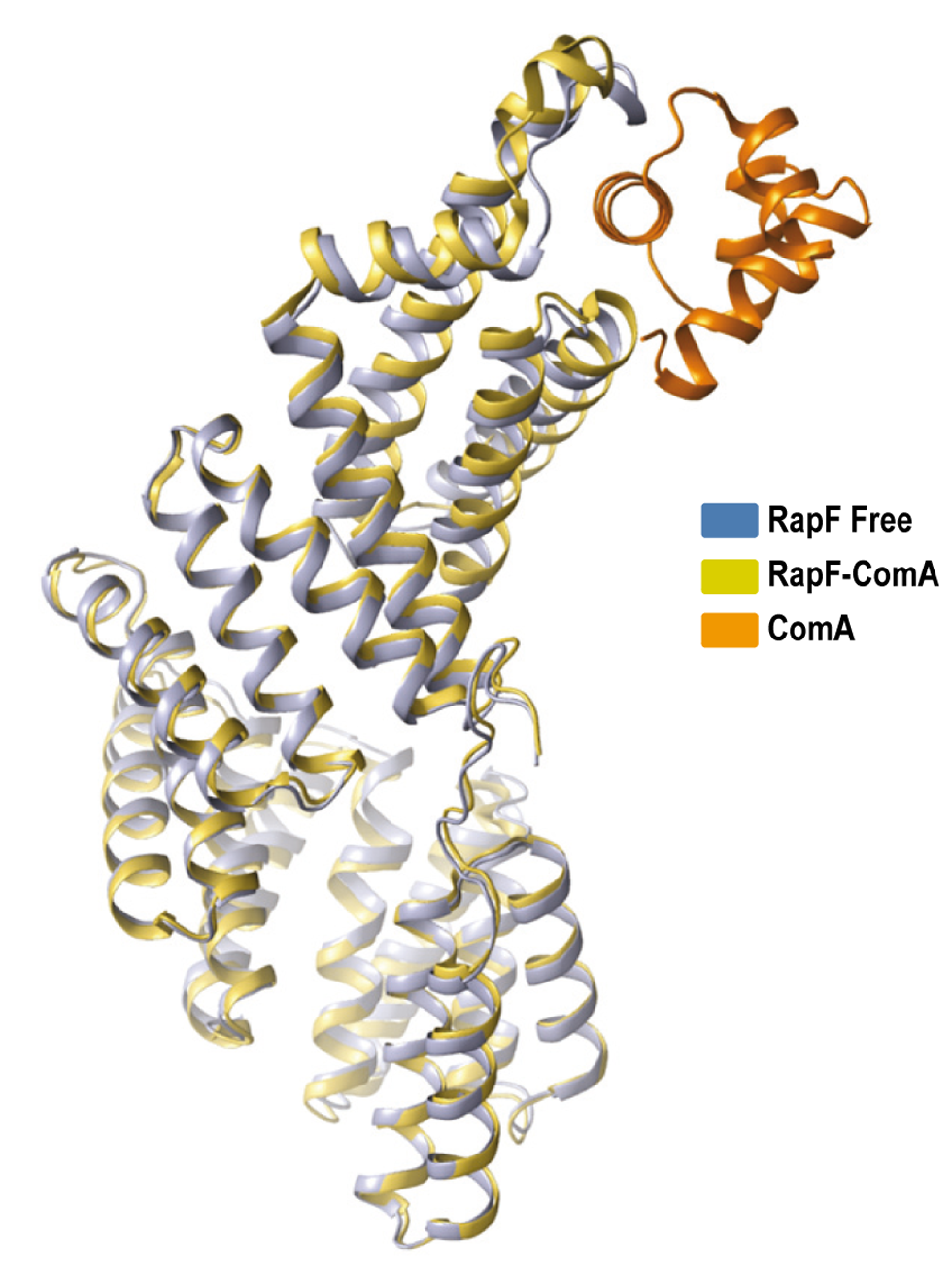

Supplement: Figure S2 — RapF free and RapF-ComA structures present similar conformation. Superimposed RapF structures in the presence (3ULQ; yellow and orange for RapF and ComA, respectively) and in the absence (light blue) of ComA binding domain. Both structures present an extremely similar conformation with local structural variability around the ComA binding site. (TIF) [file pbio.1001511.s002.tif]

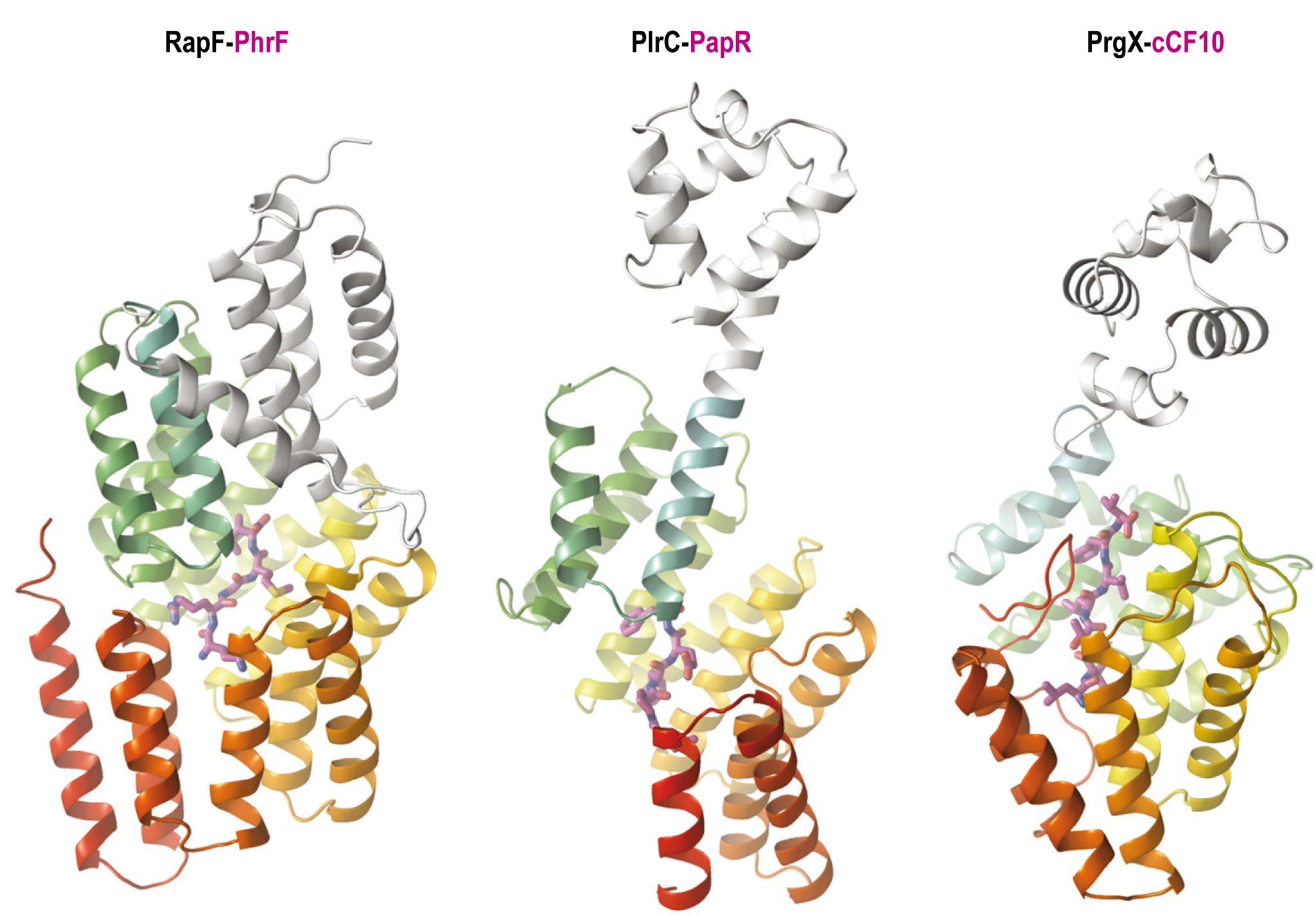

Supplement: Figure S3 — Structural comparison of RNPP family members. The structures of B. subtilis RapF-PhrF, B. cereus PlcR-PapR (2QFC) [4], and Enterococcus faecalis PrgX-cCF10 (2AXU) [9] are shown in the same view and represented in ribbon with the N-terminal effector domains colored in white and the TPR domains in rainbow. The corresponding peptides are shown in sticks rendering with carbon atoms in pink. (TIF) [file pbio.1001511.s003.tif]

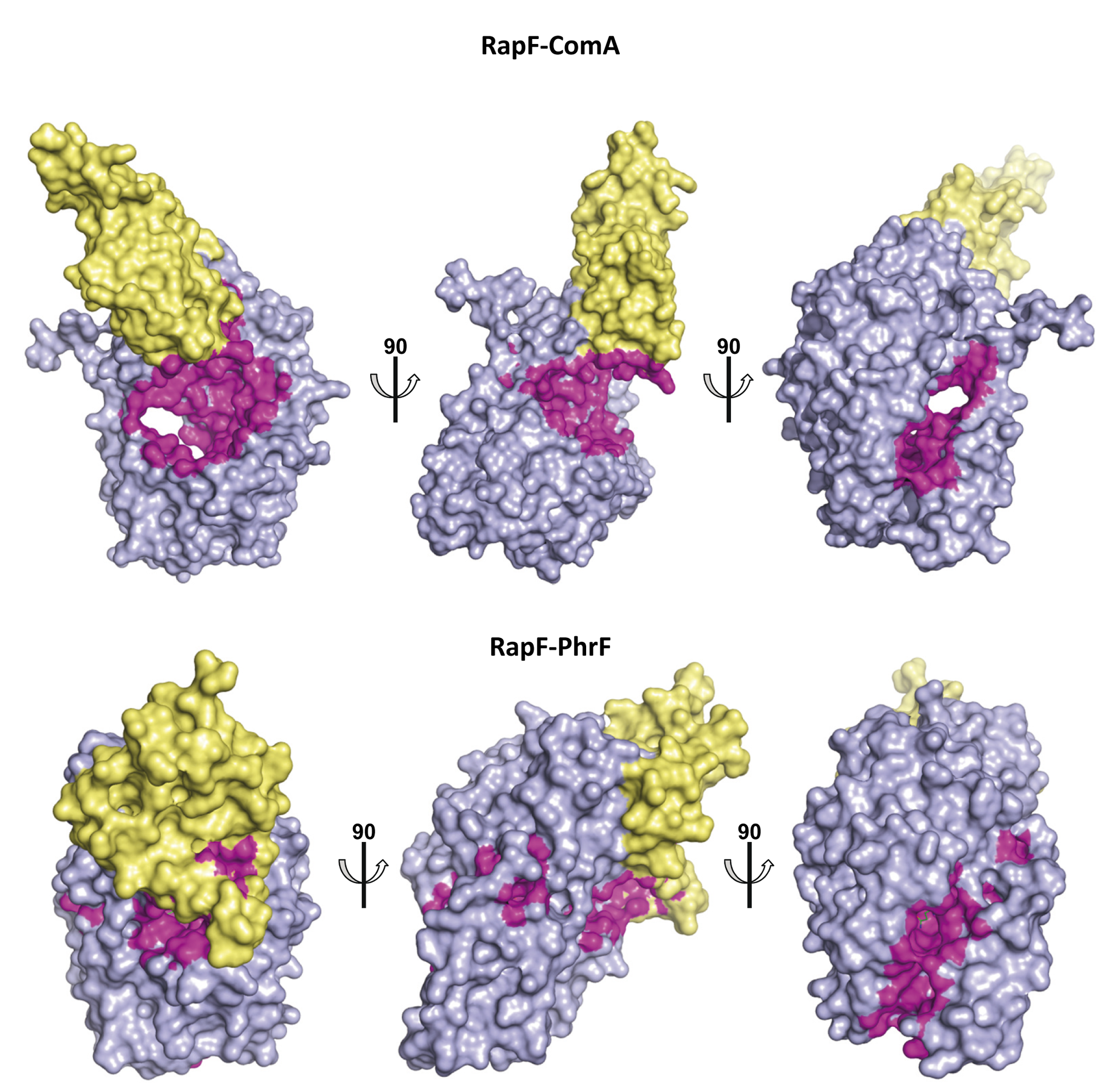

Supplement: Figure S4 — TPR channel is constricted by peptide binding. Three lateral views of RapF-ComA (upper panel) and RapF-PhrF (lower panel) structures in surface representation with N-terminal portion and TPR domain colored in yellow and blue, respectively. TPR channel surface, as is calculated by CASTp software [32], is colored in magenta. (TIF) [file pbio.1001511.s004.tif]

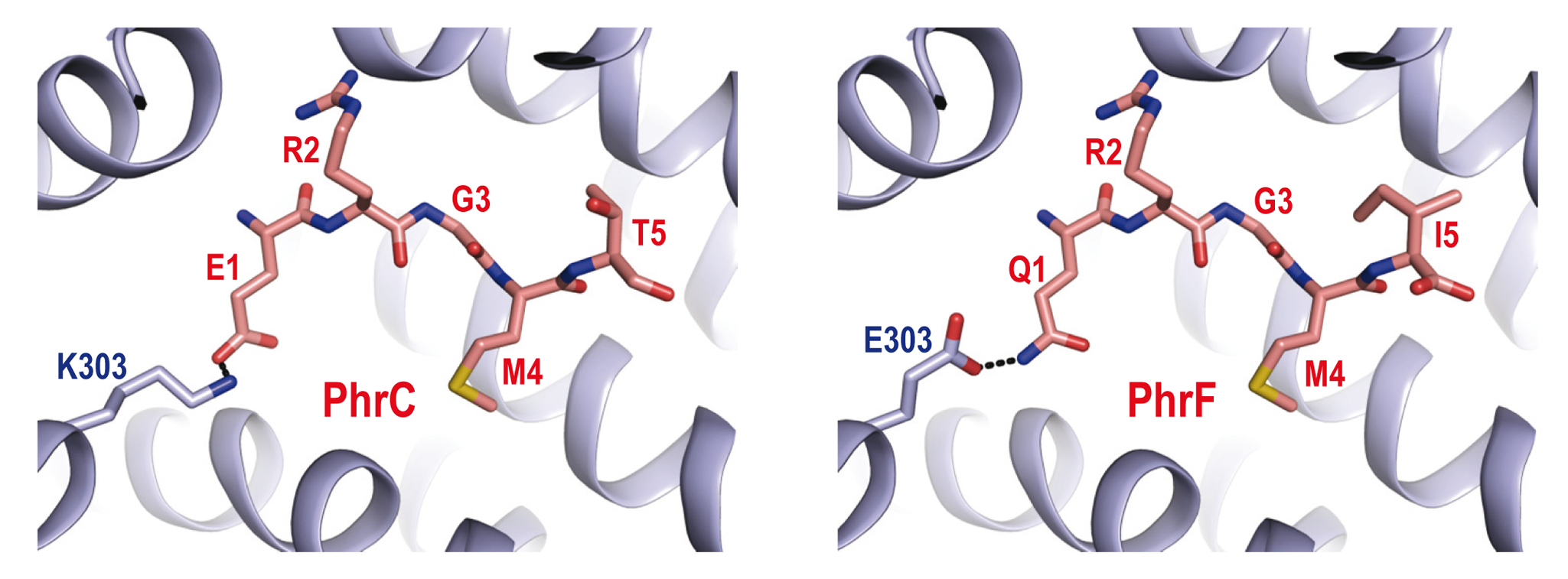

Supplement: Figure S5 — Rap peptide specificity is conferred by particular residues. Model of the close related RapC-PhrC (left) complex based in the RapF-PhrF (right) structure illustrates the structural bases of peptide specificity. Hydrogen bond between RapF Glu303 and PhrF Gln1 (showed as black dashed line) is substituted by a salt-bridged between the Lys in positions 303 of RapC and the Glu in position 1 of PhrC (black dashed line). (TIF) [file pbio.1001511.s005.tif]

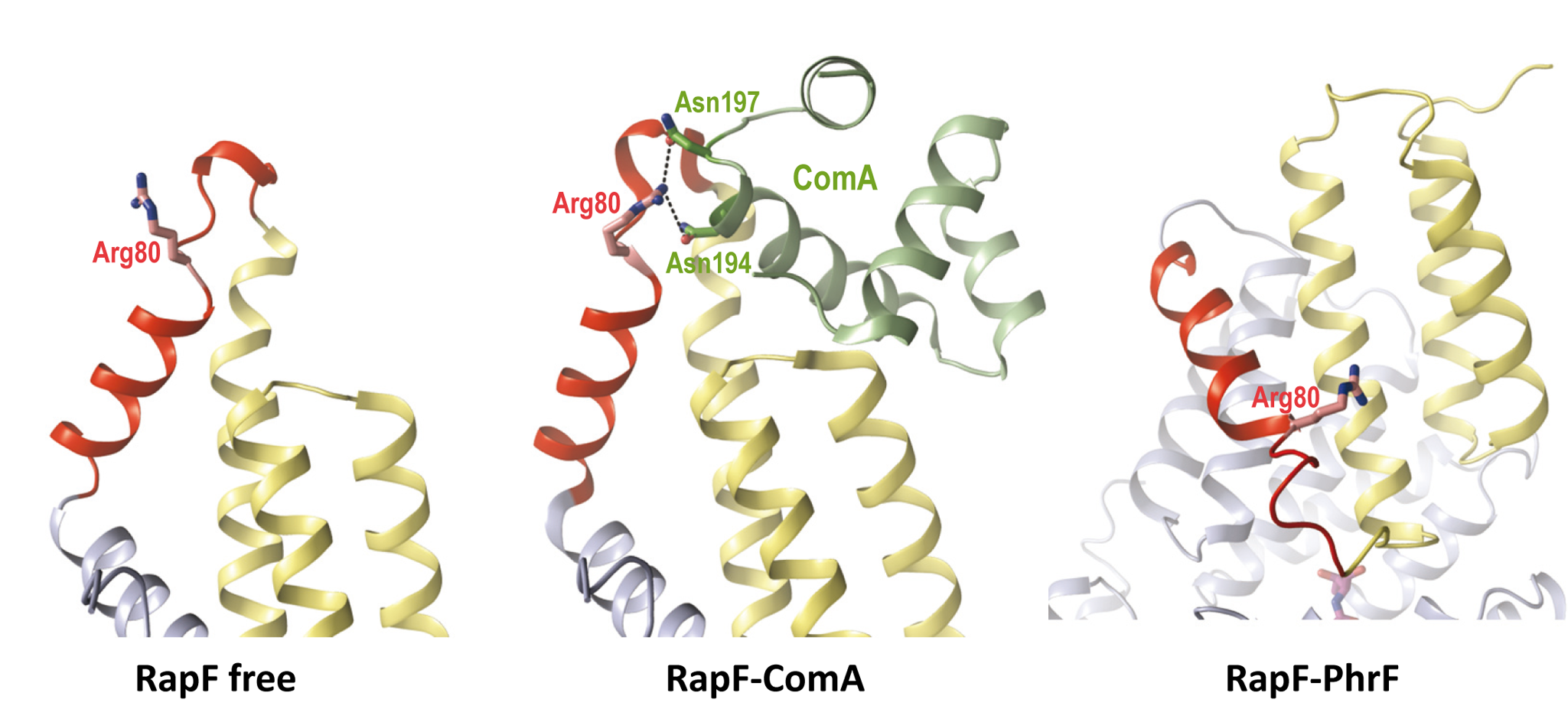

Supplement: Figure S6 — Arg80 localization in RapF structures. The position of Arg80 (sticks with carbons colored in pink) is shown in the structures of free RapF and in complex with ComA and PhrF. The 3-helix bundle, linker region and TPR domain are colored in yellow, red, and light blue, respectively. ComA is colored in green and the ComA residues interacting with Arg80 are shown in sticks with carbons in green. Arg80-ComA interactions are highlighted with dashed black lines. (TIF) [file pbio.1001511.s006.tif]
