# Supplementary material for: Structural Basis of Rap Phosphatase Inhibition by Phr Peptides
Source: PLoS Biol. 2013 Mar 19;11(3):e1001511. doi: 10.1371/journal.pbio.1001511 (PMC3601957; doi:10.1371/journal.pbio.1001511)
Supplement: Table S1 — Data collection and refinement statistics. (DOCX) [file pbio.1001511.s007.docx]

|  | **RapF-PhrF** | | | | | **RapF Free** | | | | | | |  |
| --- | --- | --- | --- | --- | --- | --- | --- | --- | --- | --- | --- | --- | --- |
|  | **Pt derivative** | | | **Native** | | **SeMet** | | | **Native** | | | |  |
| **Data collection statistics** | | | | | |  | | |  | | | |  |
| Space group | I4_1_32 | | | I4_1_32 | | P3_1_21 | | | P3_1_21 | | | |  |
| Cell parameters | a=221.96 | | | a=220.79 | | a=b=97.93  c=200.95 | | | a=b=97.2  c=203.18 | | | |  |
| Resolution (Å) ^a^ | 110-3.8  (4.01-3.8) | | | 49.3-3.1  (3.27-3.1) | | 100.5-3.2  (3.58-3.4) | | | 101.5-2.25  (2.37-2.25) | | | |  |
| Unique reflections | 9517 | | | 15637 | | 19128 | | | 42047 | | | |  |
| Completeness (%) | 99.3 (99.3) | | | 100 (100) | | 100 (100) | | | 99.6 (100) | | | |  |
| Multiplicity | 11.1 (11.3) | | | 22 (22.3) | | 7 (7.2) | | | 6.4 (7.4) | | | |  |
| I/σ | 6.2 (2.1) | | | 31.3 (3.3) | | 15.4 (3.3) | | | 6 .0 (32.1) | | | |  |
| R_pim_ (%)^b^ | 0.107 (0.351) | | | 0.021 (0.265) | | 0.073 (0.278) | | | 0.020 (0.16) | | | |  |
| **Refinement statistics** | | | | | | | | | | | | |  |
| Resolution range (Å) |  | | | 156 (3.1) | |  | | | 84.21 (2.4) | | | |  |
| R_factor_ (%)^c^ |  | | | 0.205 (0.292) | |  | | | 0.236 (0.285) | | | |  |
| R_free_ (%)^d^ |  | | | 0.239 (0.318) | |  | | | 0.258 (0.309) | | | |  |
| *B-factors (Å^2^):* |  | | |  | |  | | |  | | | |  |
| Wilson plot |  | | | 109.3 | |  | | | 47.0 | | | |  |
| Mean B_factors_ |  | | | 61 | |  | | | 48.8 | | | |  |
| Protein (MC, SC)^e^ | | | | 60.9 (58.4, 63.2) | |  | | | 48.5 (48.4, 48.6) | | | |  |
| Ligand (MC, SC) | | | | 66.2 (66.6, 65.8) | |  | | | 54.6 | | | |  |
| Water | | |  | 68.6 | |  | | | 51.1 | | | |  |
| Rms on bond distance(Å) | | 0.013 | | | | | 0.006 | | | |  | | |
| Rms on bond angles (°) | | | | 1.419 | |  | | | 0.953 | | | |  |
| *Ramachandran plot:* | | | |  | |  | | |  | | | |  |
| Most favored (%) | | | | | 92.3 | | |  | | 90.2 | |  |  |
| Additional allowed(%) | | |  | 6.5 | |  | | | 9.1 | | | |  |
| Generously allowed (%) | | |  | 1.1 | |  | | | 0.7 | | | |  |
| *Number of atoms:* | | |  | 3205 | |  | | | 6873 | | | |  |
| Protein | | |  | 3146 | |  | | | 6521 | | | |  |
| Ligands | | |  | 41 (peptide) | |  | | | 46 (sucrose) | | | |  |
| Water | | |  | 18 | |  | | | 306 | | | |  |

*^a^* The values in parentheses refer to statistics in the highest bin.

*^b^ R*_pim_=∑_h_ √(1/(n_h_−1)) · ∑_l_(|I_hl_−<I_h_>|)/ ∑ _h_∑_l_(<I_h_>)

*^c^ R*_factor_ = ∑*_h_*[*F_o_*(*h*) - *F_c_*(*h*) /∑*_h_F_o_*(*h*), where *Fo* and *Fc* are the observed and calculated structure-factor amplitudes, respectively.

*^d^ R*_free_ was calculated with 7.6% of the data excluded from the refinement.

*^e^* (MC, SC)=Main Chain, Side Chain.
