# Supplementary material for: Structural Basis of Rap Phosphatase Inhibition by Phr Peptides
Source: PLoS Biol. 2013 Mar 19;11(3):e1001511. doi: 10.1371/journal.pbio.1001511 (PMC3601957; doi:10.1371/journal.pbio.1001511)
Supplement: Table S2 — Oligonucleotides. (DOCX) [file pbio.1001511.s008.docx]

| Oligo name | Sequence (5´-3´) |
| --- | --- |
| RapF 5´ NcoI | GGCGCCATGGTGACAGGTGTC |
| RapF 3' XhoI | ATGCCTCGAGTTAGACTTCAATTTCATAC |
| RapF ^E303K^+ | ATATATTTATCAAAATTTGAATTTTTGAAATCTTTATAC |
| RapF ^E303K^- | CAAAAATTCAAATTTTGATAAATATATTACATCTCCGGC |
| Test RapF ^E303K^ | GATTTCAAAAATTCAAATTTTG |
| RapF ^E303A^+ | ATATATTTATCAGCATTTGAATTTTTGAAATCTTTATAC |
| RapF ^E303A^- | CAAAAATTCAAATGCTGATAAATATATTACATCTCCGGC |
| Test RapF ^E303A^ | GATTTCAAAAATTCAAATGCTG |
| RapF ^N227A^+ | GAACTTTGTACGCAATCGGGCTTTGTAAAAACAGCC |
| RapF ^N227A^- | CAAAGCCCGATTGCGTACAAAGTTCTCCCC |
| Test RapF ^N227A^  RapF ^D194A^+  RapF ^D194A^- | CAAAGCCCGATTGCG'  GCCACCAATTTTTTAGCGTTAAAACAGTATGAGG  CCTCATACTGTTTTAACGCTAAAAAATTGGTGGC |
| ComA 5´NcoI | GCGCCATGGAAAAGATACTAGTGATTGATG |
| ComA 3´HindIII | GTCTGCCACATGAAATTTTCGAACCGT |
